# Williams syndrome and Ebstein’s anomaly: A rare association

**DOI:** 10.4103/0974-2069.58322

**Published:** 2009

**Authors:** Vishal Changela, Sunita Maheshwari, Meenakshi Bhat

**Affiliations:** Department of Pediatric Cardiology, Narayan Hrudayalaya Institute of Cardiac Sciences, Bangalore, India; 1 Department of Medical Genetics, Narayan Hrudayalaya Institute of Cardiac Sciences, Bangalore, India

**Keywords:** Congenital heart disease, elastin haploinsufficiency, florescence *in situ* hybridization

## Abstract

We report a rare case of Williams syndrome associated with Ebstein’s anomaly of the tricuspid valve. To our knowledge, such an association has never been reported.

## INTRODUCTION

Williams syndrome is a rare genetic syndrome where 50% of the patients have associated congenital heart disease. Among a variety of lesions reported so far, the most common lesions are supra-valvar aortic stenosis and peripheral pulmonary stenosis.

We report a case of Williams syndrome with a very rare association - Ebstein’s anomaly of the tricuspid valve. This is the first report of such an association in literature and widens the spectrum of cardiac defects seen in Williams syndrome.

## CASE REPORT

**Figure 1:**
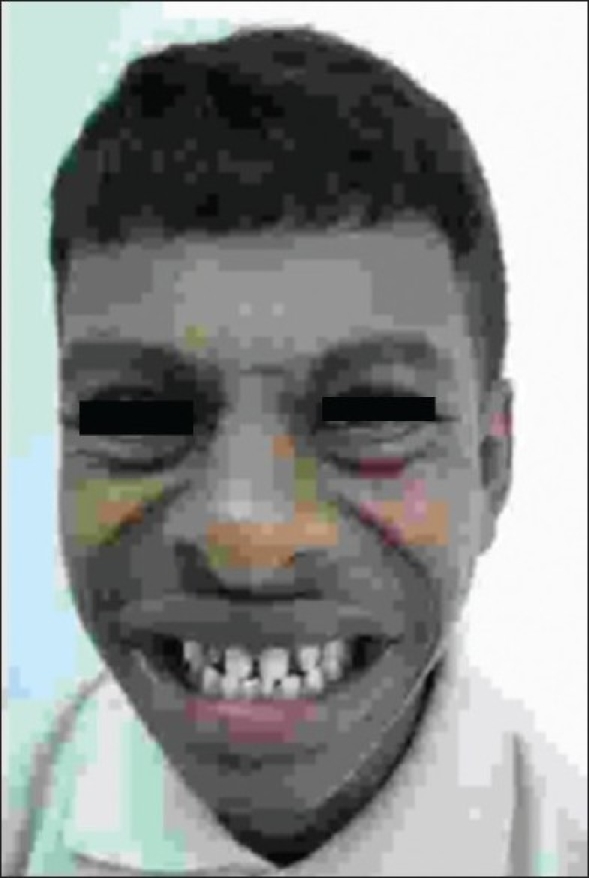
The distinctive facial features such as periorbital fullness, full cheeks with prominent naso-labial folds, thick lips, and widely spaced permanent dentition suggestive of Williams syndrome in the patient

A 15 year old boy presented in a clinic with symptoms of dyspnea (NYHA, Class I). He was short (height less than 3^rd^ centile) with global developmental delay, a very sociable personality, and a hoarse voice. A physical examination showed dysmorphic facial features like periorbital fullness, full cheeks with prominent naso-labial folds, thick lips, and widely spaced permanent dentition. Clinically, Williams syndrome was suspected [Figure 1].

Vital parameters i.e., heart rate, blood pressure, and oxygen saturation (by pulse oxymetry) were within normal limits. A cardiovascular examination revealed normal precordium without abnormal pulsations or thrill. Auscultatory findings included normal heart sounds and a pan-systolic murmur (Grade 2/6) in the tricuspid area without additional sounds and murmurs. The clinical differential diagnosis was small ventricular septal defect or tricuspid valve regurgitation.

Electrocardiogrphic features were normal sinus rhythm, QRS axis – 120 degree, normal PR interval, tall peaked *P* waves (’Himalayan’ *P* waves), and splintered polyphasic QRS complexes. It did not show features of pre-excitation. A chest X-ray revealed a cardiothoracic ratio of 0.55, right atrial enlargement, normal pulmonary blood flow, and narrow vascular pedicle.

**Figure 2:**
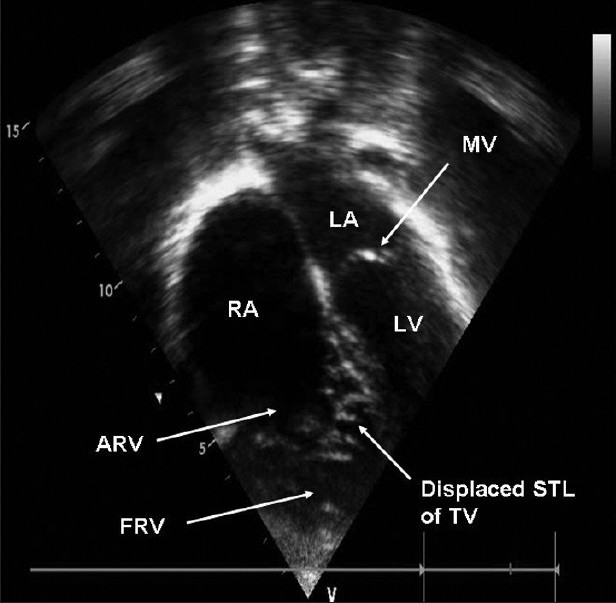
A transthoracic echocardiographic image (at apical four-chamber window) showing apical displacement of the tricuspid valve and atrialized portion of the right ventricle, which is characteristic of Ebstein’s anomaly of the tricuspid valve. (ARV - Atrialized right ventricle, FRV - Functional right ventricle, LA - Left atrium, LV - Left ventricle, MV - Mitral valve, RA - Right atrium, STL - Septal tricuspid leaflet)

A transthoracic echocardiography was diagnostic of Ebstein’s anomaly of the tricuspid valve (significant apical displacement of deformed tricuspid valve and atrialized right ventricle) and mild tricuspid valve regurgitation [Figure 2]. Other valves (mitral, aortic, and pulmonary) and cardiac structures were normal on echocardiography. The genetic diagnosis of Williams syndrome was confirmed by a florescence *in situ* hybridization test for elastin haploinsufficiency.

Orthopedic and ophthalmologic evaluations did not reveal any abnormality. Relevant normal investigations included blood glucose, serum calcium, and thyroid function test. As he did not have any significant cardiac symptoms, a regular medical follow-up was advised along with genetic counseling.

## DISCUSSION

Williams-Beuren syndrome is a multi-system developmental disorder caused by a micro-deletion of chromosome 7q11.23.[1] In virtually all cases of Williams syndrome, haploinsufficiency (loss of 1 of 2 copies) due to a deletion at chromosome band 7q11.23 that involves the elastin gene is implicated. Most deletions (over 90%) are detected through fluorescent *in situ* hybridization for a 1.5 to 2-Mb deletion.[2] It is therefore the investigation of choice in most laboratories. Absence of the elastin gene is responsible for the cardiac defects and facial dysmorphology. Other features include retardation of growth and mental development, sociable personality, visuospatial and cognitive deficits, hypercalcemia, herniae, radioulnar synostosis, and systemic hypertension.[3]

Around 53-100% of patients with Williams syndrome suffer from cardiac defects.[4‐10] Various cardiovascular abnormalities are reported in 423 patients from nine selected international series published in the last two decades. These cardiovascular abnormalities and their combined prevelance include supra-valvar, valvar, or sub-valvar aortic stenosis (72%), valvar or peripheral pulmonary stenosis (39%), systemic hypertension (17%), mitral valve prolapse (15%), coarctation of aorta (4%), bicuspid aortic valve (3%), and hypoplasia of aorta (2%). [1‐10] Other rare cardiovascular associations reported in literature are atrial and ventricular septal defects, patent ductus arteriosus, tetralogy of Fallot, endocardial cushion defect, and coronary artery stenosis.

Williams syndrome with Ebstein’s anomaly of tricuspid valve has never been reported in literature and this case widens the spectrum of cardiac defects seen in this condition.

This case highlights the need for the pediatricians and cardiologists to be aware of the fact that apart from classic supravalvar aortic stenosis and branch pulmonary stenosis, a wide range of congenital cardiac defects can occur in Williams syndrome.
